# Three-Dimensional-Printed Scaffolds for Meniscus Tissue Engineering: Opportunity for the Future in the Orthopaedic World

**DOI:** 10.3390/jfb12040069

**Published:** 2021-12-02

**Authors:** Angelo V. Vasiliadis, Nikolaos Koukoulias, Konstantinos Katakalos

**Affiliations:** 1 2nd Orthopaedic Department, General Hospital of Thessaloniki “Papageorgiou”, 56403 Thessaloniki, Greece; 2 School of Medicine, Aristotle University of Thessaloniki, 54124 Thessaloniki, Greece; 3 Orthopaedic Department, Sports Injuries Unit, Saint Luke’s Hospital, 55236 Thessaloniki, Greece; nkoukoulias@yahoo.gr; 4 Laboratory for Strength of Materials and Structures, Department of Civil Engineering, Aristotle University of Thessaloniki, 54124 Thessaloniki, Greece; katakaloskostas@gmail.com

**Keywords:** meniscal tissue engineering, meniscal regeneration, 3D printing, scaffold, biomaterials

## Abstract

The meniscus is a critical component of a healthy knee joint. It is a complex and vital fibrocartilaginous tissue that maintains appropriate biomechanics. Injuries of the meniscus, particularly in the inner region, rarely heal and usually progress into structural breakdown, followed by meniscus deterioration and initiation of osteoarthritis. Conventional therapies range from conservative treatment, to partial meniscectomy and even meniscus transplantation. All the above have high long-term failure rates, with recurrence of symptoms. This communication presents a brief account of in vitro and in vivo studies and describes recent developments in the field of 3D-printed scaffolds for meniscus tissue engineering. Current research in meniscal tissue engineering tries to combine polymeric biomaterials, cell-based therapy, growth factors, and 3D-printed scaffolds to promote the healing of meniscal defects. Today, 3D-printing technology represents a big opportunity in the orthopaedic world to create more specific implants, enabling the rapid production of meniscal scaffolds and changing the way that orthopaedic surgeons plan procedures. In the future, 3D-printed meniscal scaffolds are likely to be available and will also be suitable substitutes in clinical applications, in an attempt to imitate the complexity of the native meniscus.

## 1. Introduction

**Figure 1:**
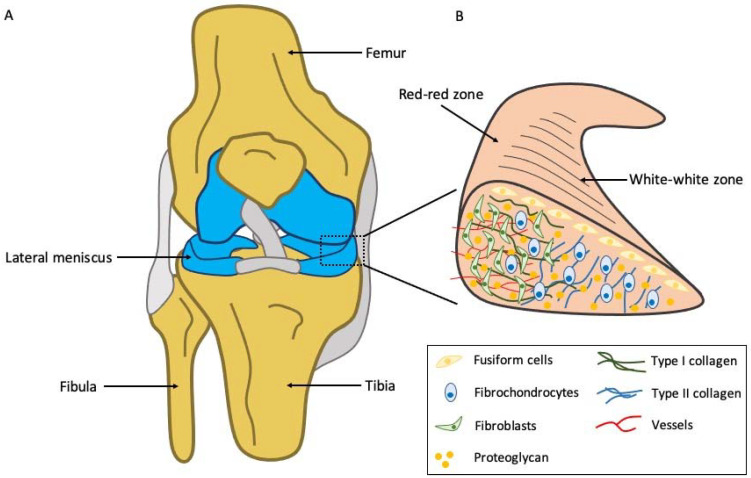
Basic anatomy of the knee, with the meniscus located within the knee joint between the tibial plateau and the femoral condyles (**A**), which consists of distinct zones of varying cell populations that are distinct in morphology and phenotype; the inner region contains rounded or oval shaped fibrochondrocytes that produce type II collagen, whereas the outer region is mainly populated by fibroblast-like cells and randomly oriented type I collagen. Aggrecan, which is the major proteoglycan, is organized in a complex architecture and provides the tissue-specific biomechanical characteristics (**B**).

The knee is one of the largest and most complex joints in the human body. Every structure in the knee joint is important, while the meniscus represents a bulwark against the destruction of the joint. The meniscus is a fibrocartilaginous structure, which is found within the knee joint between the medial and lateral femoral condyles and the tibia (Figure 1) [1]. As a vital part of the joint, it plays a significant role in shock absorption, by distributing evenly the load between the medial and lateral compartment and increases the joint congruity and diffusion of synovial fluid along the articular surfaces during physical activity [1,2]. The knee joint still represents the most common anatomical location of pain seen in primary care or general practice settings [3]. In fact, meniscal injuries are known to be the most frequently encountered and treated injuries in the knee joint and, as a result, their prompt diagnosis and appropriate management are of great importance in orthopaedic practice [1,2,4].

In this communication, we will mainly focus on the options provided by 3D-printing technology and the combination of different materials, in order to build a 3D construct similar to a native meniscus, giving the opportunity to change the direction of treatment in the near future.

Non-operative management is useful for the initial treatment of meniscal injuries. Anti-inflammatory and analgetic drugs, strengthening exercises of the quadriceps, modifications in activities of daily living, unloader bracing, and intra-articular injections have been shown to improve knee function and reduce joint pain [1]. In the case of failure of non-operative management, there are three main methods for the operative management of meniscal tears (from resection to preservation): (i) arthroscopic total/partial meniscectomy, (ii) meniscal repair, and (iii) meniscal reconstruction (meniscal scaffolds, meniscal allograft transplantation) [1,5,6]. Although, meniscal transplantation is generally utilized as an alternative management option for selected patients, with previous complete or almost-complete meniscectomy, it is a technically demanding and time-consuming procedure. Therefore, it should be performed only after considerable practice and taking into consideration factors related to the patient (skeletally mature, mild unicompartmental degenerative changes, younger than 45 years, normal mechanical axis of the knee joint) [1,7].

## 2. Principles of Meniscal Substitution

There are three types of meniscus replacement and reconstruction: (i) autografts (tendon, fat or/and perichondral tissue), (ii) allogenic transplants (cadaveric), and (iii) artificial meniscus prostheses [8]. Autografts use a patient’s own tissue, with no immunologic reactions. Recently, surgeons have started using tendon autografts to replace the meniscus, with several advantages: (i) the possibility of replacing the entire meniscus, (ii) availability of any size of meniscus, and (iii) relatively fast patient recovery [8,9,10]. Autografts are intended to stimulate the native cell migration, to promote the remodeling phase, and resulting in the formation of meniscus-like tissue [8]. Meniscal allografts may be considered as a preferred modality for knee joint restoration. However, allografts have many limitations, such as mismatch between donor and recipient (difficulty in obtaining a matching allograft meniscus); the availability of donor tissue in many countries; the technically demanding operation, which can take several hours to perform; its longevity and possibility of disease transmission [8,9]. As a result, three-dimensional (3D)-printed biomaterial constructs offer an effective way to achieve improved biomimetic strategies and have been tested to generate different human tissues, including meniscus [2].

In the field of tissue engineering and regenerative medicine, 3D-printing technology has grown in the last decades, starting from 1986, in order to fabricate customized objects without the need for a mold [11,12]. The first 3D-printing machine was patented by researchers at the Massachusetts Institute of Technology in 1994 and become commercially available in 1997 [12]. The application of 3D-printing facilities has shown promising results for quick and cost-effective solutions in different medical fields, such as cardiothoracic surgery, neurosurgery, urology, dentistry, vascular, and orthopaedic surgery [6,11,13].

The advantages of using 3D-printing technology include the ability to product patient-specific scaffolds with complex shapes, which are capable of homogenous cell distribution, and the ability to imitate the extracellular matrix [14,15,16]. However, it should be noted that they have serious disadvantages, such as the need for specialized equipment, the expensive materials, and the production time needed for scaffolds with a more precise and intricate construction (Table 1) [16,17]. In order to overcome these issues, researchers use natural and synthetic polymers (Table 2), or their combination, as engineered scaffolds and have demonstrated their promising properties for meniscal regeneration [2,9,18].

## 3. Applications of 3D-Printing Scaffolds for Meniscal Tissue Engineering

**Figure 2:**
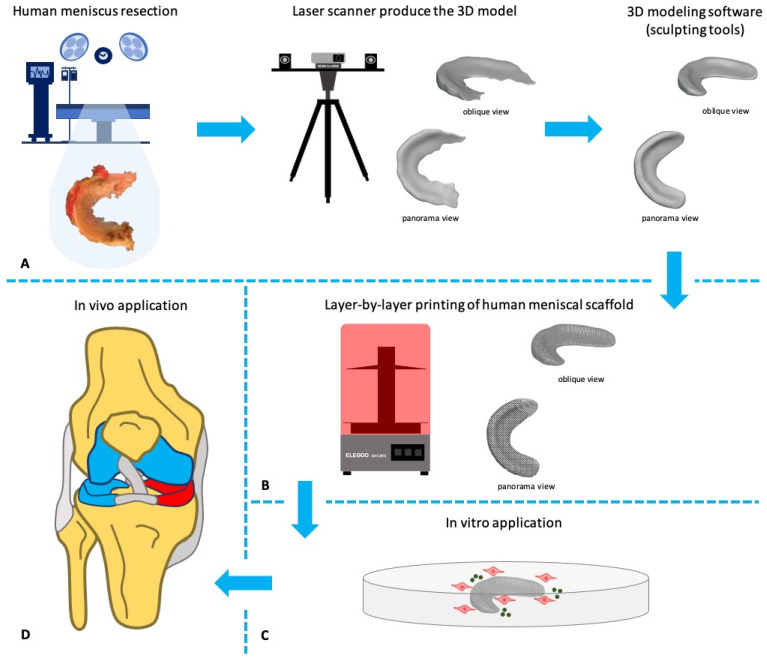
The cycle of scaffold fabrication and implantation. The anatomic contour of the medial meniscus was obtained from a live human donor, who had undergone total knee replacement, and a custom-made meniscal scaffold was fabricated for a total graft (**A**), with layer-by-layer deposition of silicone resin using 3D printing technology (**B**). The meniscal scaffold was bioactivated with primary or stem cells and growth factors, and cultured in a bioreactor, stimulating at least one aspect of the in vivo environment (**C**). The final step was the meniscal scaffold implantation (**D**).

Considering the field of orthopaedics, it is possible to identify four main application categories of 3D-printing technology: (i) surgical pre-operative planning, (ii) patient-specific implants and orthoses, (iii) personalized instruments and surgical guides, and (iv) biomaterial constructs (tissue-specific scaffolds or/and small tissues) [6,19]. The 3D-printed meniscal scaffolds are a very promising technology, which it is capable of manufacturing patient-specific scaffolds with custom shapes and consistent quality [20]. The development of 3D-printing has provided a new horizon for meniscus repair and regeneration and, as a result, there is a clear need to develop patient-specific implants (Figure 2).

A semi-automatic segmentation method was proposed, in order to obtain a 3D model of the meniscus tissue made from polycaprolactone (PCL) with different internal architectures. For this reason, high-quality MRI images were acquired from healthy male volunteer subjects and an advanced segmentation software was used for the fabrication of tissue engineering scaffolds [21]. This 3D model is a vital step in the right direction to the translation of individualized tissue engineering into daily clinical practice, where treatment of meniscal lesions is envisioned. Filardo et al. developed a patient-specific meniscus prototype based on 3D-bioprinting of a human cell-laden scaffold. A 3D model of a bioengineered medial meniscus was created, based on MRI scans of a human volunteer. Primary mesenchymal stem cells were isolated from bone marrow of the iliac crest and used for cell-laden bio-ink preparation. They found that the selected bio-ink presented good printability and shape fidelity, allowing the fabricated tissue to mimic the morphology of the native meniscus [22]. The fabrication of a 3D cell-printed meniscus construct presents great potential as an advanced strategy for the effective repair of the damaged meniscus [18,22]. For example, Chae et al. developed a 3D cell-printed meniscus scaffold using a mixture of synthetic polymers and cell-laden decellularized meniscal extracellular matrix bio-ink [18]. In vivo experiments demonstrated that the 3D cell-printed meniscus scaffolds exhibited biocompatibility and excellent mechanical properties as well as improved biological functionality [18].

PCL is a synthetic, biodegradable polymer that has been widely used in biomedical applications, because of its high biocompatibility and mechanical strength [20,23]. In tissue engineering, a 3D-printing-based biomimetic and composite tissue-engineered meniscus scaffold, consisting of PCL/silk fibroin (SF) was evaluated and demonstrated high biocompatibility and biomechanical properties. This combination of PCL and an SF scaffold augmented by synovium-derived mesenchymal stem cells demonstrated excellent structural, biomechanical, and functional properties for meniscus regeneration and chondroprotection [23]. However, using PCL scaffolds in axial tensile tests, would lead to deformation at different rates and may create a break in the structure, which renders it unsuitable for translation applications. In order to overcome this, Gupta et al. developed a composite scaffold, which mimics the mechanical properties of the human meniscus and also presents a self-healing capacity, leading to the repair of microfractures during loading and unloading cycles. They utilized a 3D-printed scaffold and an interpenetrating network (IPN) based on a hydrogel system and achieved retention of functionality of the differentiated chondrocytes within the IPN and formation of the vasculature of the transplanted scaffold [24].

The engineering of human meniscus remains challenging, due to its two district regions: the outer vascularized dense fibrous connective tissue, and the inner completely avascular and aneural fibrocartilage region, with a high proportion of proteoglycans, which are responsible for the viscoelastic compressive and the hydration grade. [7,9,24,25]. Hydrogel-based biomaterials play a pivotal role in meniscal engineering strategies, due to their high water content, which resembles the meniscal tissue, and due to the fact that they can be fabricated under favorable conditions, enabling the encapsulation of cells and labile biomolecules [25]. Hydrogel-based biomaterials have the ability to be seamlessly integrated into water matrices and create a more ‘native’ microenvironment; making them the first choice for 3D bioprinting applications for meniscal repair [26].

While conventional hydrogels are highly biocompatible and widely used for tissue engineering processes, they present certain disadvantages, such as high cost, possible immunogenic responses, and low mechanical resistance; restricting their use in persistent load-bearing applications (i.e., the knee joint) [27]. Therefore, hydrogels can be programmed through simple chemical modification (reduction-oxidation reactions, ions, protonation, and the cleavage of acid–labile chemical bonds) to exhibit optimal properties, such as porosity, biodegradability, and biocompatibility, in order to prevent an excessive immune response, while reducing the reliance on immune suppression [28,29]. Moreover, electrospinning is an attractive method for creating nanofibrous scaffolds, in order to reinforce the poor mechanical properties of hydrogels [30].

In more recent studies, different human cell sources have been used in conjunction with electrospun scaffolds and hydrogel, as an attractive combination for generating meniscus-like neotissue [31,32]. In one study, Baek et al. used human cells, which were seeded onto aligned electrospun collagen type I scaffolds and were encapsulated in a tricomponent hydrogel [31]. They found that multilayered constructs composed of electrospun scaffolds, with infrapatellar fat pad cells embedded in a tricomponent hydrogel, produced the most meniscus-like neotissues, with greater deposition of collagen type I, while generating the highest mechanical properties compared to the meniscal, mesenchymal stem, and synovial cells [31]. Romanazzo et al. developed a biomimetic construct that can instruct encapsulated stem cells to differentiate into either meniscal chondrocytes or fibroblasts [32]. They found that an alginate hydrogel functionalized with an extracellular matrix derived from inner and outer region of the meniscus was able to differentiate into either meniscal chondrocytes or fibroblasts. They also exhibited the printability of these functionalized hydrogels, demonstrating that their co-deposition alongside PCL micro-filaments improved the mechanical properties of the 3D bioprinted constructs [32].

Interestingly, Bahcecioglu et al. fabricated a 3D-printed, artificial and anatomically-shaped meniscus scaffold, and subsequently infused it with two different cell-laden hydrogels in the outer and inner part, to imitate the bizonal biochemical composition of the meniscal [33]. In their study, a meniscal construct with total variation was produced by using agarose and gelatin methacrylate hydrogels in the inner and outer region of the 3D-printed PCL scaffolds, and which was cartilage-like and fibrocartilage-like at the inner and outer portion, respectively [33]. This technique has potential for future use as a substitute for total meniscal replacement.

Despite the promising prospects of tissue-engineered scaffolds for meniscus regeneration, there are still many important problems with the use of current materials to build a construct similar to a native meniscus [20,22,23]. The meniscus extracellular matrix (MECM) is usually derived from xenogeneic and allogeneic tissue sources, with a possible negative reaction provoking strong immune and toxicity responses [20]. A possible explanation for this immune response may be the inadequate decellularization of the source tissue. For that reason, Chen et al. combined a MECM-based hydrogel and 3D-printed scaffold to stimulate whole meniscus regeneration [34]. In their study, a hybrid, 3D-printed and wedge-shaped porous PCL scaffold, followed by injection with the optimized MECM-based hydrogel, promoted whole meniscus regeneration in vivo [34]. In a recent in vitro and in vivo study, performed by Sun et al., a ready-to-implant anisotropic meniscus, utilizing a 3D-bioprinting protein releasing a cell-laden hydrogel-PCL composite scaffold, was evaluated by transplanting it into the knee joints of goats [35]. This 3D-bioprinted meniscal scaffold contained mesenchymal stem cells (MSCs), cell-laden hydrogel encapsulating PLGA microparticles carrying transforming growth factor-beta 3 (TGF-β3), or connective tissue growth factor (CTGF) in different regions. The in vitro and in vivo evaluation of the meniscus constructs demonstrated resemblance to the native meniscus, as well as long-term chondroprotection of the regenerated meniscus [35].

## 4. Conclusions

The use of 3D-printing in the orthopaedic field needs to be taken into consideration, in order to change health care. The advent of 3D-printing technology, with new 3D imaging techniques, makes the near future of meniscal scaffolds promising; while personalized 3D meniscal scaffolds are starting to be designed. Today, similarly to any new technology, 3D-printing meniscal scaffolds have introduced many advantages and possibilities to the orthopaedic world, and they provide a big opportunity to help and change the way that orthopaedic surgeons plan their procedures. In particular, hydrogels have been recognized as promising vehicles for tissue engineering and play an important role in 3D bioprinting. Thus, the ideal hydrogel-based scaffolds should provide safety and biocompatibility, be easily manufactured, be flexible in application, and should possess suitable pores for cell adhesion, growth, and proliferation. Despite this progress, the properties of hydrogel-based scaffolds can be more improved by combining different natural or synthetic polymers and using 3D-printing technology to manufacture the meniscus. Finally, legislative regulations and rules must be established, in order to ensure that meniscal scaffolds meet the appropriate criteria and to guarantee their correct use.

## Figures and Tables

**Table 1:** Summary of the advantages and disadvantages of 3D-printing scaffolds in the field of tissue engineering and regenerative medicine.

3D-Printing Scaffolds for Meniscus Tissue Engineering	
Advantages	Fabrication of complex structures
	Use of various types of biomaterials
	Easy application of computer-assisted methods
	Scaffold design using patient-specific data
Disadvantages	Specialized equipment
	Expensive materials
	Production time (more precise and intricate scaffold)
	Highly specific protocols

**Table 2:** Summary of natural and synthetic polymers for meniscus tissue engineering.

Polymeric Materials		Types
Natural polymers	Proteins	Collagen
		Silk fibroin
		Gelatin
	Polysaccharides	Hyaluronic acid
		Sodium alginate
		Agarose
		Chitosan
Synthetic polymers	Aliphatic polyesters	Polylactic acid (PLA)
		Polycaprolactone (PCL)
		Polylactic-co-glycolic acid (PLGA)
		Polyglycolic acid (PGA)
	Others	Polyurethane (PU)
		Polyethylene glycol (PEG)
		Polycarbonate urethane (PCU)
		Polyvinyl alcohol (PVA)
		Polyethylene oxide (PEO)

## Data Availability

The data presented in this study are available on request from the corresponding author.
